# Changes in ocular aquaporin-4 (AQP4) expression following retinal injury

**Published:** 2008-09-25

**Authors:** Adnan Dibas, Ming-Hui Yang, Shaoqing He, Joseph Bobich, Thomas Yorio

**Affiliations:** 1 Department of Pharmacology and Neuroscience, University of North Texas Health Science Center at Fort Worth, TX; 2 Department of Chemistry, Texas Christian University, Fort Worth, TX

## Abstract

**Purpose:**

Changes in the expression of water channels or aquaporins (AQP) have been reported in several diseases. However, such changes and mechanisms remain to be evaluated for retinal injury. This study was designed to analyze changes in the expression of AQP4 following elevation of intraocular pressure (IOP) and after intravitreal endothelin-1 injection and the potential involvement of the ubiquitin-dependent proteasome.

**Methods:**

Retinal injuries were induced by the elevation of intraocular pressure in rat eyes using the Morrison model or following endothelin-1 intravitreal injection. Immunohistochemistry using a combination of glial fibrillary acidic protein (GFAP) and aquaporin-4 antibodies were employed to follow changes in the optic nerve head astrocytes. Real-time quantitative PCR (Q-PCR) was used for measuring changes in AQP4, ubiquitin hydrolase L1 (UCH-L1), and β-actin messages. Changes in AQP4, caspase-3, thy-1, ubiquitination, and GFAP expression were also followed in total retinal extracts using western blotting. An S5a column was used to purify ubiquitinated proteins.

**Results:**

In retinas of both injury models, there was an upregulation of GFAP (a marker of astrogliosis), caspase-3, and downregulation of thy-1, a marker for retinal ganglion cell stress, and decreased retinal AQP4 mRNA and protein levels as determined by Q-PCR, and western blotting, respectively. By contrast, IOP enhanced expression and co-localization of GFAP and AQP4 in optic nerve astrocytes. AQP4 was detected in affinity-purified ubiquitinated proteins using S5a column, suggesting that AQP4 is a target for degradation by the ubiquitin-dependent proteasome. While elevation of IOP induced an increase in ubiquitination in retinal extracts, it decreased ubiquitination in optic nerve extracts as detected by western blotting. Enhanced ubiquitination and decreased ubiquitination appear to correlate with AQP4 expression. IOP decreased UCH-L1 (or protein gene protein [PGP9.5]) in retinal extracts as judged by Q-PCR.

**Conclusions:**

The enhanced expression of AQP4 in optic nerve astrocytes following elevation of IOP may explain the astrocytic hypertrophy normally seen in glaucoma patients and may involve alteration in the activity of ubiquitin-dependent proteasomal degradation system. The decreased ubiquitination in the optic nerve may lead to increased levels of proapoptotic proteins known to be degraded by the proteasome, and thus to axonal degeneration in glaucoma.

## Introduction

The glaucomas represent a heterogeneous group of diseases that result in a progressive optic neuropathy characterized by functional and structural impairment of ocular tissues. Particularly affected are the trabecular meshwork, the optic nerve head, and retinal ganglion cells.

One of the risk factors in primary open angle glaucoma (POAG) is an associated elevation in intraocular pressure (IOP) [1]. Elevation of IOP results in blockade of axonal transport in animals as well as displacement of the optic nerve head [2–7]. In addition, high levels of endothelin-1 (ET-1) have been observed in patients with normal tension glaucoma, open angle glaucoma, and animal models of glaucoma [8–11]. Low doses of ET administered intravitreally have produced similar ocular nerve damage, optic nerve cupping, and inhibition of anterograde axonal transport in the optic nerve [12–15]. Abnormalities in water balance play an important role in the pathophysiology of a variety of neurologic disorders. Neuronal activity is associated with redistribution of water. While shrinkage of the extracellular space around active synapses is observed, enlarged extracellular space volume at distal sites also occurs [16].

The discovery of aquaporins (AQPs) has provided a molecular basis for understanding water transport in several tissues, including the ocular system [17]. At least six AQPs are expressed in the eye: AQP0 major intrinsic protein (MIP) in lens fiber; AQP1 in cornea endothelium, ciliary and lens epithelia, trabecular meshwork, and retinal photoreceptor cells; AQP3 in conjunctiva; AQP4 in ciliary epithelium and retinal Muller cells; AQP5 in corneal and lacrimal gland epithelia [18]; and AQP9 in retinal ganglion cells [19].

Hypoxia, ischemia, hyperosmotic stress, and elevated ET levels are associated with changes in the densities of AQP expression. Interestingly, such insults are key risk factors in the development of glaucomatous optic nerve neuropathy. However, the expression of ocular aquaporins upon retinal injuries has not been fully characterized. In addition, the hallmark of glaucoma is the demyelination and degeneration of the optic nerve and death of retinal ganglion cells, leading eventually to irreversible blindness. Axonal degeneration in many neurodegenerative diseases (e.g., Alzheimer and Parkinson diseases) appears to be mediated via abnormalities in the activities of the ubiquitin-proteasomal-degradation system (UPS). Elevated IOP has been found to block both anterograde and retrograde axonal transport in the optic nerve head in animals [2–7] which, in turn, may impede the anterograde transport of ubiquitin in the optic nerve axons [20], and thus impair the proteasomal function known to control the levels of several pro-apoptotic proteins [21]. In addition, ubiquitin hydrolase L1 (UCH-L1) activity and expression has been shown to correlate with axonal degeneration. Using two models of retinal injuries: 1) intravitreal injection of ET-1; and 2) elevation of IOP according to the Morrison procedure, changes in AQP4 and ubiquitination levels were evaluated in retinal and optic nerve extracts.

## Methods

### Materials

Anti procaspase-3 antibodies were from Cell Signaling (Boston, MA) and monoclonal anti-thy-1 antibodies were from Chemicon, (Temecula, CA). AQP4 (mouse and rabbit) and thy-1 rabbit antibodies were from Santa Cruz Inc (Santa Cruz, CA) and monoclonal anti-glial fibrillary acidic protein antibodies were from Neomarkers (Fremont, CA). Monoclonal anti-tubulin antibodies were from Upstate Inc (Lake Placid, NY), and secondary antibodies (donkey anti-mouse conjugated Alexa 633, goat anti-rabbit conjugated Alexa 633, and goat anti-mouse conjugated Alexa 488) were from Invitrogen Inc (Carlsbad, CA).

### Intravitreous injection of ET-1

HEPES-buffered ET-1 or HEPES vehicle buffer alone were injected intravitreally as described [15]. Male Brown Norway retired breeder rats were anesthetized and 4 µl of HEPES-buffered ET-1 (2 nmol) or HEPES vehicle, was injected into the vitreous of the left eye with a 30-gauge needle attached to a syringe (µl 710, 22s gauge; Hamilton Co., Reno, NV) by polyethylene tubing (PE-20, Clay Adams Brand; BD Biosciences, Sparks, MD). Rats were anesthetized with intramuscular cocktail (xylazine/ketamine/acepromazine). Anesthesia cocktail for sedation (9 ml) was prepared as follows: 5 ml ketamine (stock 100 mg/ml) + 0.5 ml xylazine (stock 100 mg/ml) + 1 ml acepromazine (stock 10 mg/ml) + 2.5 ml sterile water. The animal is given 1 ml/kg weight. During intravitreous injections, retinas were observed through the pupil with a surgical microscope (model Stiffuss S; Carl Zeiss, Thornwood, NY). During introduction of solution into the vitreous, a transient blanching of the retina was observed in all animals. One minute after injection, all retinas appeared normal in color. All studies were conducted in accordance with National Institutes of Health guidelines, the ARVO Statement for the Use of Animals in Ophthalmic and Vision Research, and guidelines of the University of Texas Health Science Center Committee on Animal Welfare. After 48 h, 72 h, or 7 days post ET-1 injection, all rats were euthanised by an i.p. injection of pentobarbital (100 mg kg^−1^), eyes were dissected and processed for western blotting as described in 2.5 or for Q-PCR as described in 2.6.

### The Morrison glaucoma model

Male Brown Norway retired breeder rats weighing 200-300 g were used and fed standard chow. Prior to experimentation, the animals were housed individually in rat gang cages in an environmentally controlled room and maintained on a 12 h:12 h light-dark cycle and allowed tap water ad libitum. IOP was elevated in one eye, by following the description by Morrison et al. [22] in which 50 μl of 1.8 M saline was injected into the episcleral veins of one eye of anesthetized rats such that blanching was observed. This procedure produces scarring of the trabecular meshwork (TM) with a resultant rise in IOP and damage to the optic nerve [23]. Rats were housed post-surgically in constant low light (<90 lux) to minimize effects of circadian influences on IOP. After the rats were stabilized and conscious, IOP measurements were taken with a Tonolab rebound tonometer (Colonial Medical Supply Co. Inc., Franconia, NH) after 0.1% proparacaine, a topical anesthesia, was applied. Animals were weighed periodically. IOP was initially monitored twice a week for up to 10–14 days and subsequently monitored on a weekly basis, once the IOP was greater than 25% of baseline IOP values. IOP values at each time were recorded as an average of nine consecutive Tonolab measurements for each eye. Mean intraocular pressure changes over the time of pressure elevation were calculated for each eye. IOP exposure is the cumulative IOP difference between the hypertensive (treated) eye to control (normotensive) eye and was calculated for each animal as mmHg days as previously described by McKinnon et al. [24]. Accordingly, IOP exposure for each rat was calculated by performing separate area-under curve (AUC) integration of IOP over the days of exposure for the treated and control eye. The integral value of the control eye was subtracted from that of the experimental treated eye to yield the “IOP-integral difference” and was expressed as mmHg days. Rats with elevated IOP were maintained for 500–700 mmHg days post-surgery. Comparisons between groups of various mmHg days of IOP elevation were made using one-way ANOVA and multiple comparison tests at p *<*0.05. Rats (n=3) were also injected with isotonic saline (sham) into one eye and kept under the same conditions as those that received hypertonic saline for 140 days. Rat eyes were dissected, and either fixed in formalin for immunohistochemistry or processed for western blotting or Q-PCR. The contralateral eye served as the control.

### Double immunofluorescence labeling studies

Rat eyes were dissected, fixed in formalin, embedded in paraffin, and 5 µm sections were obtained using a microtome. Retina sections were deparaffinized in xylene, rehydrated using ethanol washes, and washed with PBS (150 mM NaCl, 3.8 mM NaH_2_PO_4_, 16.2 mM Na_2_HPO_4_, Sigma-Aldrich Co. St. Louis, MO). Permeabilization was done with 0.1% Triton X-100 and PBS washes before nonspecific binding was blocked by 5% BSA in PBS. The sections were incubated for 2 h at room temperature with a proper mixture of primary antibodies: 1:400 thy-1, 1:100 AQP4, and 1:100 GFAP. Coverslips were rinsed with PBS three times and allowed to incubate for 1 h in the dark at room temperature in a mixture of proper secondary antibodies: donkey antimouse conjugated Alexa 633, goat antirabbit conjugated Alexa 633, and 1:400 goat antimouse conjugated Alexa 488. This was followed by three washes in 1X PBS and one final wash in deionized water. Coverslips were mounted on glass slides in antifade medium (FluorSave; Calbiochem, La Jolla, CA) and allowed to dry for 20 min in the dark. Retinal and optic nerve sections were viewed and images were taken with Zeiss LSM 410 confocal microscope. Control was performed by omitting primary antibodies.

### Western blotting

Western blotting was performed on either total retinal lysates or enriched plasma membrane and cytosolic fractions. Total retinal lysate was prepared by dissecting retinas from eyes and solubilized them into a 250 μl of a solution containing 20 mm Tris (pH 7.4), 10% sucrose, 2 mm EDTA, 2 mM EGTA, 50 mM NaF, 1% Triton X-100, 0.1% sodium dodecyl sulfate, and protease inhibitors at 4 °C. Retinal lysates were incubated for 30 min on ice then briefly sonicated before centrifugation at 14,000x g for 15 min. The supernatant was collected and protein concentration was measured using a bicinchoninic acid (BCA) protein assay kit (Sigma, St. Louis, MO) with BSA as the standard. Plasma membrane fractions were enriched by dissecting retinas from eyes and homogenized them 4 °C , using a Potter homogenizer (60 strokes), into 700 μl of a solution containing 20 mm Tris (pH 7.4), 10% sucrose, 2 mm EDTA, 2 mM EGTA, 50 mM NaF, and protease inhibitors. This was followed by centrifugation at 3,000x g for 5 min. The unbroken tissue was sonicated 7 times then centrifugation was repeated. The retinal supernatant was then centrifuged for 30 min at 100,000x g at 4 °C. The resultant supernatant (enriched cytosol) and pellet (enriched plasma membrane) were collected for protein measurement using the BCA protein assay. Optic nerves, 1.5 cm in length, were removed and homogenized in 100 μl of BUST sample buffer: 2% β-mercaptoethanol, 5 M urea, 1% SDS, 0.1 M Tris, and 0.02% bromophenol blue, pH 7.4. Proteins were separated by 10% sodium dodecyl sulfate-PAGE, with 20–30 μg (enriched fraction) or 100 μg (total retinal extract) of protein loaded in each lane. Gels were equilibrated for 10 min at room temperature in a transfer buffer: 192 mM glycine, 20% methanol and 25 mM Tris-HCl, pH 8.3. Afterwards, gels were electroblotted on nitrocellulose membranes for 75 min at 100 V using a Bio-Rad electroblotting unit. The membranes were dried at room temperature. Western blotting was performed using Amersham Chemiluminescent Kit (Bedford, MA). Membranes were incubated with 1 μg/ml primary antibodies for 60 min and with 1:10,000 secondary antibody for 30 min . Next, membranes were exposed to an X-ray film for 30 s, 1 min, and 3–5 min, and then the film was later developed. Afterwards, membranes were stripped and probed with anti-α-tubulin antibodies for normalization. Experiments were repeated three times. Band densities were quantified with image-analysis software (Scion, Frederick, MD), and the intensity of AQP4/thy-1/GFAP/caspase-3 bands was normalized for every sample relative to the intensity of the respective tubulin bands.

### Real-time analyses of AQP4/UCH-L1/β-actin mRNA levels

Retinas were dissected from eyes, and total RNA was extracted with Trizol (Life Technology, Carlsbad, CA) by following the manufacturer’s instructions. In this study, 5 μg of total RNA was reverse transcribed using the iscript kit (Bio-Rad, Herculez, CA) according to manufacturer’s instructions. Control Q-PCR reactions were performed in the absence of cDNA templates. β-actin was used as a housekeeping gene. The primers for retinal AQP4 described in Table 1 and primer optimization described in Table 2. The melting curves were generated to detect the melting temperatures of the specific products immediately after the PCR run. The relative mRNA levels were determined by the comparative C_T_ (cycle number at threshold) method as described in PE Biosystems User Bulletin #2. The fold change was determined by the following formula:

**Table 1:** Quantitative PCR primer sequences and expected product sizes.

**Primer name**	**Forward primer (5’- 3’)**	**Reverse primer (5’-3’)**	**Product size (bp)**
AQP4	CGGTTCATGGAAACCTCACT	CATGCTGGCTCCGGTATAAT	191
UCH-L1	CTAGGGCTGGAGGAGGAGAT	CCCAATGGTACCACAGGAGT	189
β-actin	TGTGATGGTGGGAATGGGTCAG	TTTGATGTCACGCACGATTTCC	514

Quantification of relative RNA concentrations was achieved by using the comparative C_T_ method using β-actin gene as reference.

**Table 2:** Primer optimization.

**Primer name**	**Denaturation**	**Annealing**	**Extension**	**Number of cycles**
AQP4	95°C 30 s	60^o^C 30 s	72^o^C 60 s	45
UCH-L1	95^o^C 30 s	60^o^C 30 s	72^o^C 60 s	40
β-actin	95^o^C 60 s	60^o^C 60 s	72^o^C 120 s	40

All primers were optimized for annealing temperatures and cycles.

Change = 2 − Δ(ΔC_t_),

where ΔC_t_ = C_ttarget_ − C_tβ-actin_, and “target” is the gene of interest. The first delta is the difference between the ΔCt values of the treated eye and the control eye (ΔΔCt) and represents the corrected shift of the analyzed gene.

To verify sequence of products, we performed regular PCR and ran the PCR products on a 1.5% agarose gel in parallel with 100 bp DNA markers before staining with ethidium bromide. Bands were cut and sequenced to verify identity. The authenticity of PCR products was confirmed using a BLAST search of the sequence through National Center for Biotechnology Information.

### Statistical analyses of data

Data are reported as means±SEM. All experiments were repeated at least three times with up to three to four replicates per condition each time. Statistical significance was determined by using one-ANOVA and the Tukey multiple comparison tests at p<0.05.

## Results

**Figure 1:**
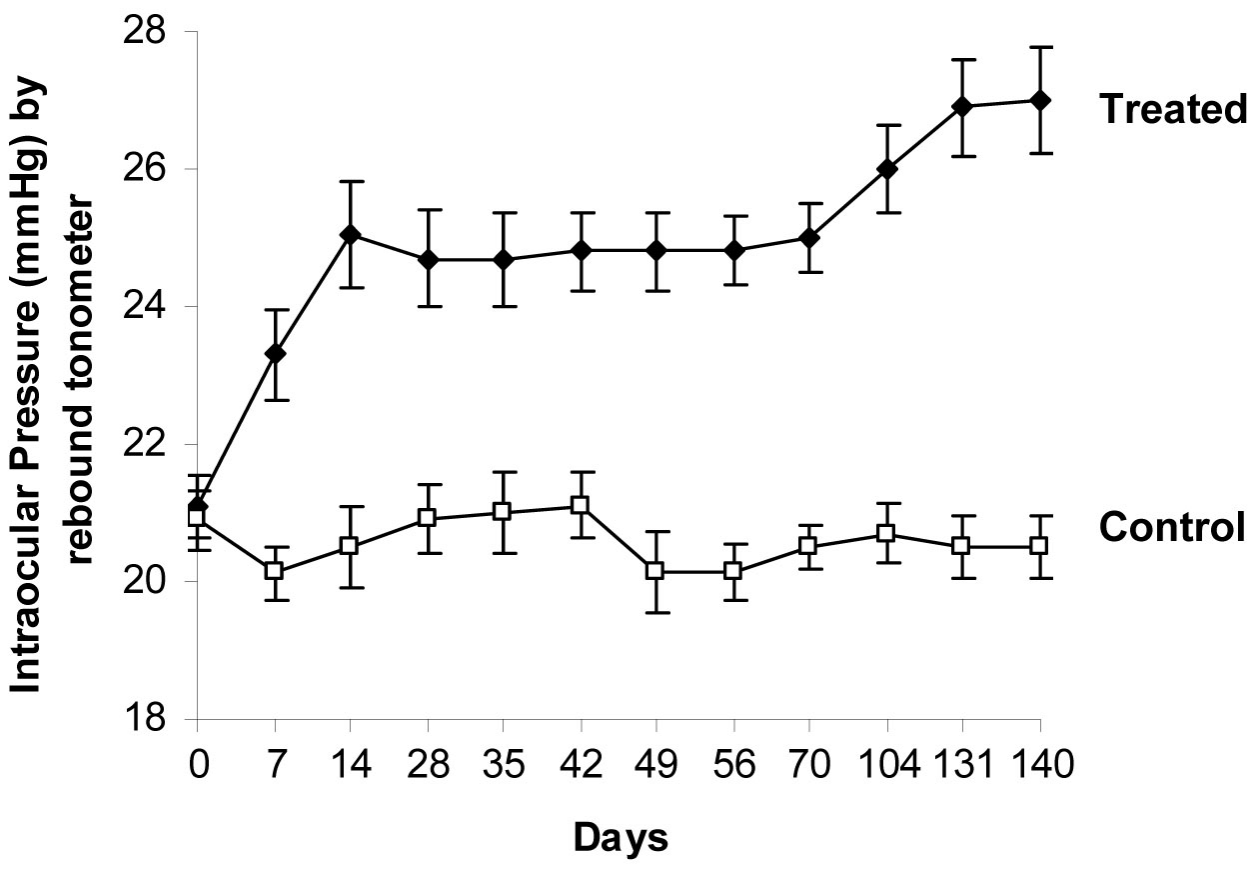
Introcular pressure measurements in a Morrison rat model of glaucoma. Hypertonic saline was given in one eye of each Brown Norway rat to elevate intraocular pressure (IOP) and the contralateral eye was allowed to serve as a control. Each data point represents the average of nine consecutive IOP measurements (mean±SEM) for each eye in mmHg IOP values.

As shown in Figure 1, IOP significantly increased in eyes with episcleral vein injection of hypertonic saline compared to the contralateral eyes and was maintained for up to 140 days post-surgery. The experimental glaucomatous eyes showed a significant and sustained increase in the IOP during the experimental period. Two days before surgery, the average baseline IOP for both control and treated eyes was 21±0.5 mmHg. However, the average IOP in the treated eye increased and peaked (days varies between animals) at 27±0.7 mmHg. Fortune et al. [25] reported an estimated 6 mmHg difference between control and elevated IOP eyes following similar surgery. While IOP readings in the current study were obtained using the Tonolab, earlier studies have reported higher IOP readings of up to 35–50 mmHg using the Tonopen XL. Moore et al. [26] have reported that the Tonopen XL significantly overestimates IOP in animals, and Pease et al. [27] suggested that the Tonolab is superior to Tonopen XL in measurement of IOP in rat and mouse eyes.

Similar observations have been cited with human glaucoma patients. For example, Weisberg et al. [28], Skaf et al. [29], and Haus et al. [30] reported higher IOP readings when using the Tonopen XL in glaucoma patients compared to readings obtained using the rebound tonometer ICT and other instruments, and suggested that measurements are probably falsely elevated when using the Tonopen XL. Isotonic saline-injected (sham) rats, maintained for up to 140 days post-injection, showed no increase in IOP. AUC measurements, yielding the IOP-integral difference of IOP elevation over days of exposure for treated eye and control eye, were calculated for each rat and represented as mmHg days. Experimentally IOP-elevated Brown Norway rats with IOP corresponding to 610±102 mmHg days were analyzed in the study.

### Effect of intravitreal injection of ET-1 and elevated IOP on AQP4 in rat retinas

**Figure 2:**
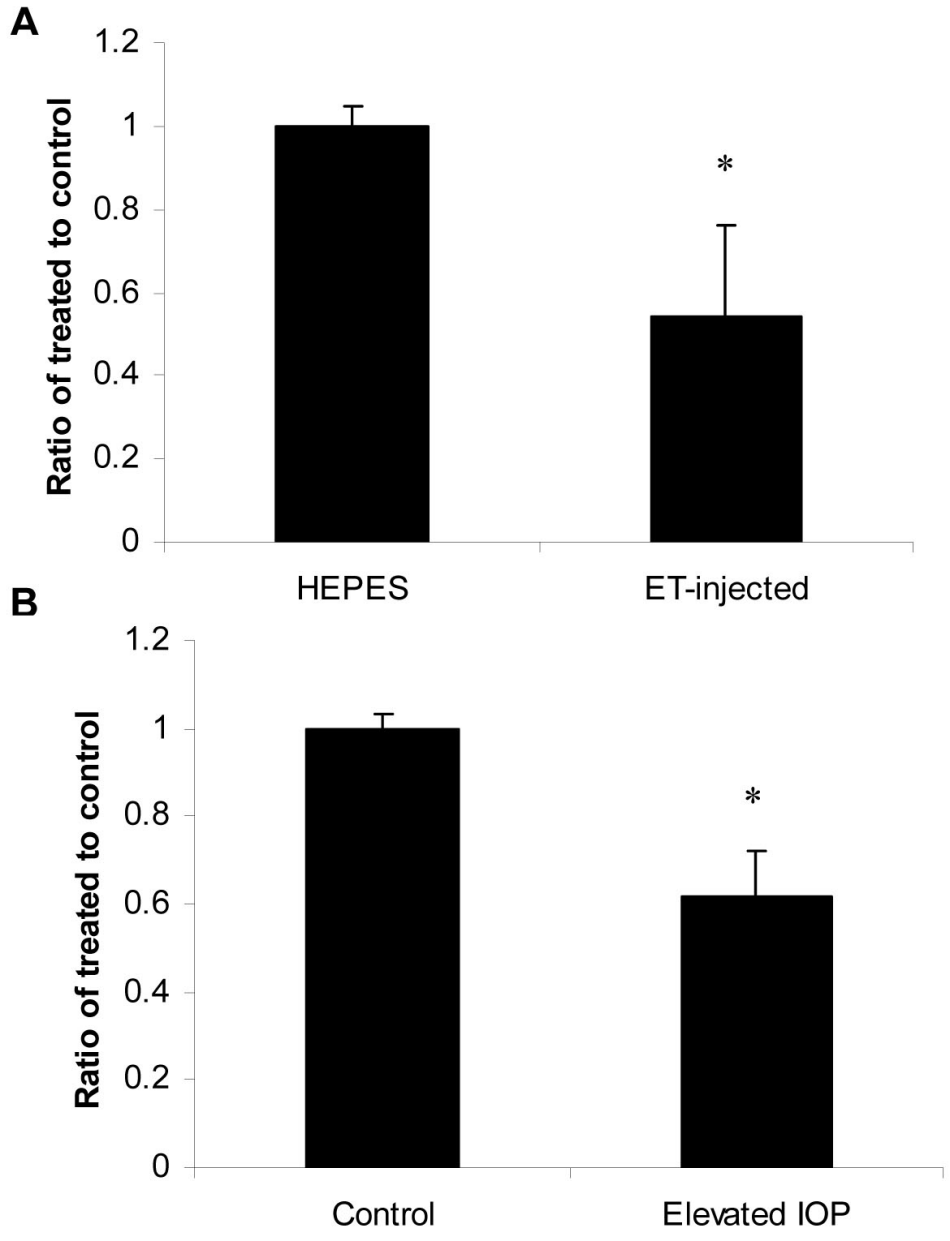
A single intravitreous ET-1 injection and elevated intraocular pressure reduced AQP4 mRNA in rat retinas. Four microliters of HEPES-buffered ET-1 (2 nmol) or HEPES vehicle, was injected into the vitreous of the left eye and 2 days later, retinas were removed, total RNA was isolated and transcribed into cDNA. IOP was elevated using the Morrison Method [22]. mRNAs for AQP4 transcripts were significantly lower compared to controls following 2 days of ET-injection (~ 45%, **A**). Elevation of IOP also decreased AQP4 transcripts by ~ 33%, as determined by quantitative real-time PCR (**B**). Gene expression data of AQP4 is calculated after normalizing with β-actin. Data are expressed as ratio of control±SEM and the asterisk denotes a significant difference compared with control-retinas at p<0.05.

**Figure 3:**
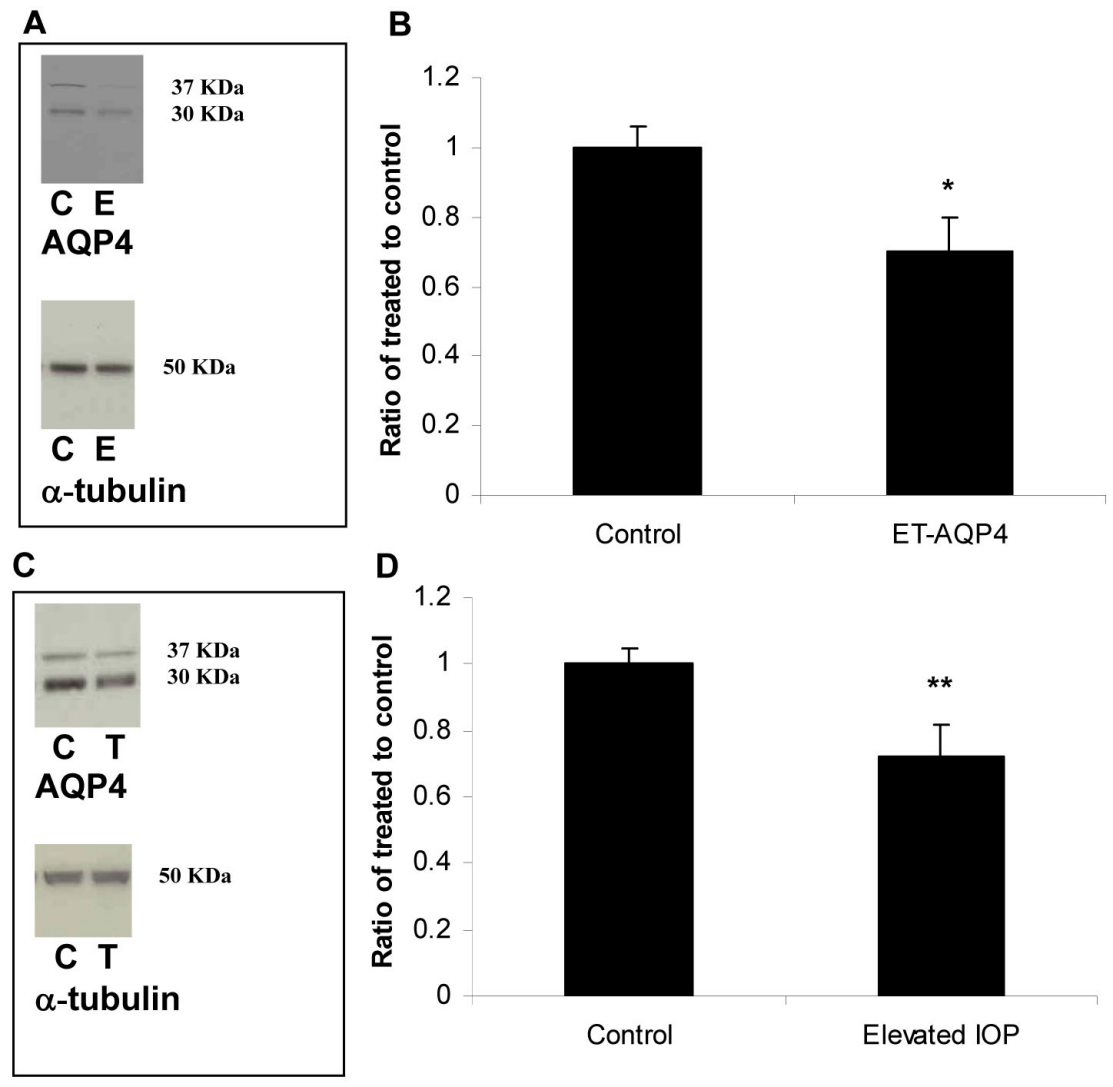
Intravitreal ET-1 injection and elevated intraocular pressure reduced AQP4 protein levels in rat retinas. Following retinal injuries with ET-injection or elevation of IOP, retinas were dissected and plasma membrane proteins were isolated. Thirty microgram protein was loaded into each lane. **A:** Immunoreactive bands for AQP4 and α-tubulin 2 days after intravitreal injection of ET-1 showing a significant reduction in AQP4 protein levels. Also, quantitative measurement using western blot showed that elevation of IOP decreased AQP4 protein levels by ~ 30% (**C**). Densitometric quantification is shown in **B** and **D**. Data are expressed as a ratio of the control value and each column represents mean±SEM. The asterisk denotes statistical significance of AQP4 in ET-injected retinas versus control (p<0.05) and the double asterisk denotes statistical significance of AQP4 in elevated IOP-retinas versus control (p<0.001) as determined by one-way ANOVA and Tukey multiple comparison test. Abbreviations: control eye (C), ET-injected eye (E), and elevated IOP (T).

Several studies have shown that ET-1-injection induced hypoxia to the optic nerve, astrogliosis and axonal loss [8–15]. Therefore, the effect of intravitreal injection of ET-1 on AQP4 expression in retina was tested. As shown in Figure 2A, ET-1 injection resulted in a decrease in AQP4 mRNA as determined by Q-PCR. Five out of 7 rats (71%) showed a significant reduction in mRNA (54±11%, p<0.001 versus HEPES-injected or control, Figure 2A), and there was a similar reduction in AQP4 mRNA upon elevation of IOP in six out nine rats (67%, 62±12, p=0.001, Figure 2B). A similar reduction was observed in AQP4 protein levels in ET-1 injected and IOP-elevated eyes. As shown in Figure 3A, nine out of 11 rats (82%, Figure 3A) showed a decrease in AQP4 protein levels in ET-1 injected eyes (70±10%, p<0.05) and AQP4 protein levels decreased in five out seven rats following elevation of IOP (71%; 72±12% p<0.001, 3C).

**Figure 4:**
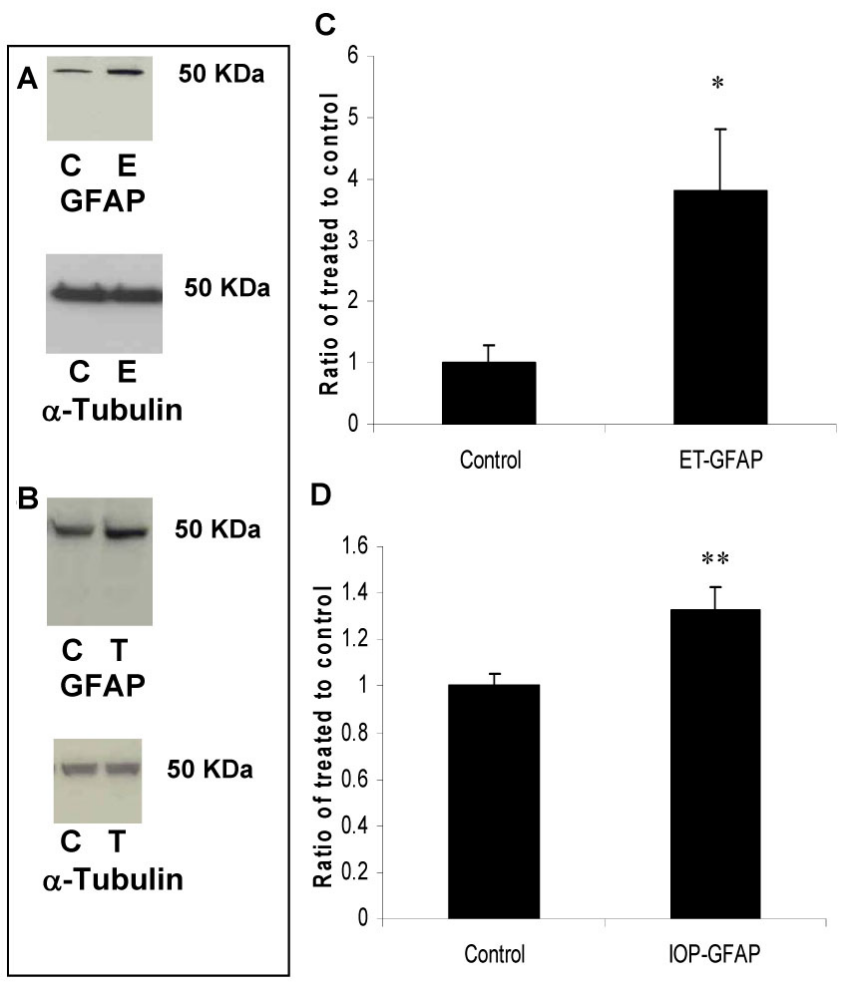
Glial fibrillary acidic protein (GFAP) protein is upregulated following intravitreal ET-1 injection and elevation of intraocular pressure. Following retinal injuries with ET injection or elevation of IOP, retinas were dissected and cytosolic proteins were isolated. Fifty microgram protein was loaded into each lane. GFAP, a cellular marker for retinal injury, was upregulated in both injury models but with significant induction following ET injection (380+100%) compared to elevation of IOP (133+10%). Densitometric quantification is shown in **C** and **D**. Data are expressed as a ratio of the control value and each column represents mean±SEM. The asterisk denotes statistical significance of GFAP in ET-injected retinas versus control (p<0.005) and the double asterisk denotes statistical significance of GFAP in elevated IOP-retinas versus control (p<0.005), as determined by one-way ANOVA and Tukey multiple comparison test. Abbreviations: control eye (C), ET-injected eye (E), and elevated IOP (T).

**Figure 5:**
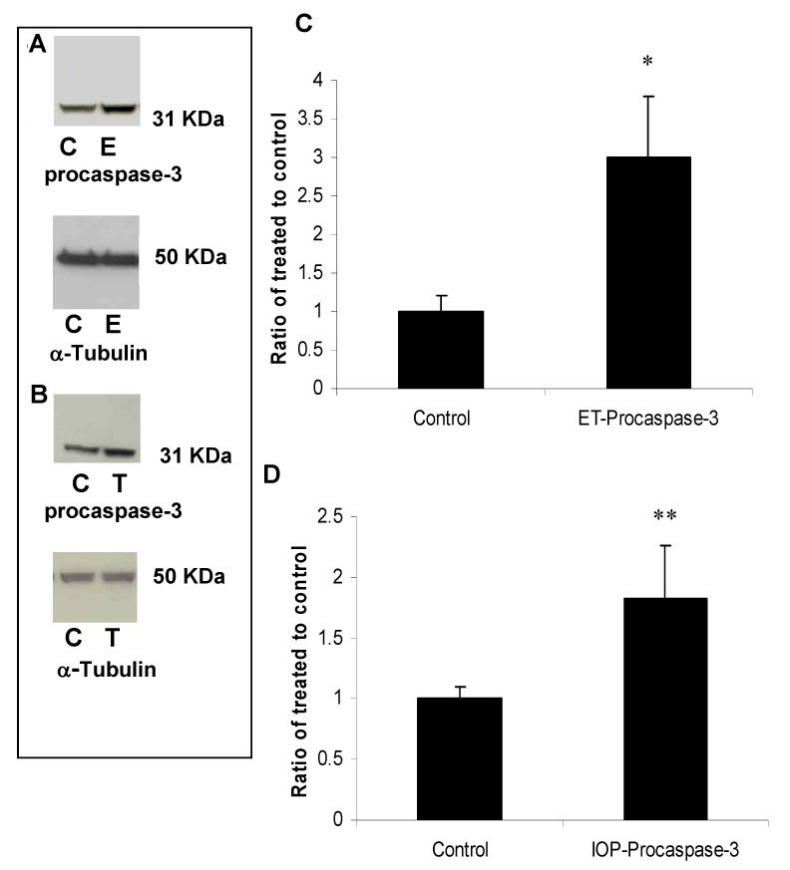
Procaspase-3 protein is upregulated following intravitreal ET-1 injection and elevation of intraocular pressure. Procaspase-3 (31 kDa) is normally cleaved to a 17 kDa fragment that is fully active. However, in both models, an upregulation of the procaspase-3 form was observed without detection of the 17 KDa fragment. **A:** Immunoreactive bands for procaspase-3 and α-tubulin 2 days after intravitreal injection of ET-1 showing a significant increase in procaspase-3 protein levels (300%). **B:** Quantitative measurement using western blot also showed that elevation of IOP increased procaspase-3 protein levels by ~ 180%. Isolated cytosolic proteins (50 μg) were loaded into each lane. Densitometric quantification is shown in figures **C** and **D**. Data are expressed as a ratio of the control value and each column represents mean±SEM. The asterisk denotes statistical significance of procaspase-3 in ET-injected retinas versus control (p<0.05) and the double asterisk denotes statistical significance of procaspase-3 in elevated IOP-retinas versus control (p<0.05), as determined by one-way ANOVA and Tukey multiple comparison test. Abbreviations: control eye (C), ET-injected eye (E), and elevated IOP (T).

**Figure 6:**
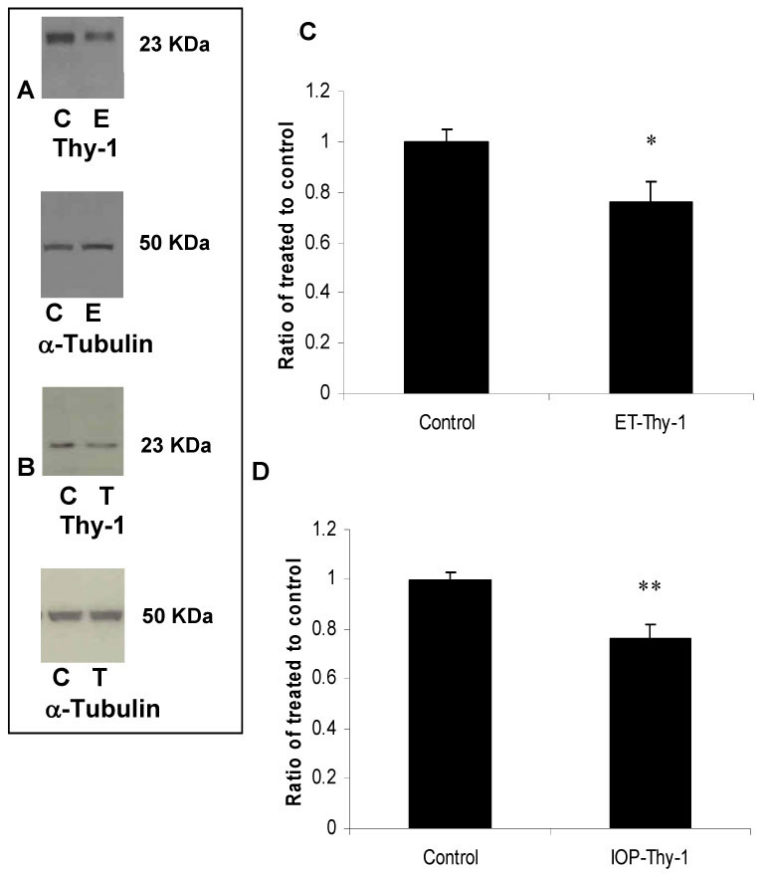
A single intravitreous ET-1 injection and elevation of intraocular pressure decreased thy-1 protein levels in rat retinas. Thy-1 expressed by retinal ganglion cells is widely used as marker for RGC early stress and was shown to be reduced following ET injection and elevation of IOP by ~ 25%. Data are expressed as a ratio of the control value and each column represents mean±SEM. Densitometric quantification is shown in **C** and **D**. The asterisk denotes statistical significance of thy-1 in ET-injected retinas versus control (p<0.05) and the double asterisk denotes statistical significance of thy-1 in elevated IOP-retinas versus control (p<0.05) as determined by one-way ANOVA and Tukey multiple comparison test. Abbreviations: control eye (C), ET-injected eye (E), and elevated IOP (T).

ET-1 injection also resulted in an increase in GFAP (Figure 4A, 380±100%, p<0.005) and similarly, elevation of IOP increased GFAP expression in all screened rats (133±10%, p<0.005, Figure 4B,C, n=6). Caspase-3 upregulation is involved in initiating apoptosis and not surprisingly was induced in both injury models. ET-injection increased procaspase-3 protein levels (300±80%, p<0.05, Figure 5A, n=6) and high IOP also increased procaspase-3 levels (180±40%, p<0.05, Figure 5B, n=6). By contrast, in both models, there was a reduction in thy-1 protein levels, a marker for RGC stress (Figure 6A,B, respectively, n=6; 75±10%, p<0.05 in both models).

### Immunohistochemical analyses of AQP4 in Morrison rat model of glaucoma

The rat optic nerve head (ONH) possesses similarities to the human ONH and can be classified into three regions [31]: the neck region, located at the level of the sclera; the transition region, an extension zone below the neck region; and the posterior region, which is characterized by myelinated axons. Astrogliosis characterized by increased labeling of GFAP and altered extracellular matrix proteins expression is observed in glaucomatous ONH [32,33]. However, it is not presently known if AQP4 expression is augmented in ONH astrocytes exposed to IOP elevation that results in hypertrophy.

**Figure 7:**
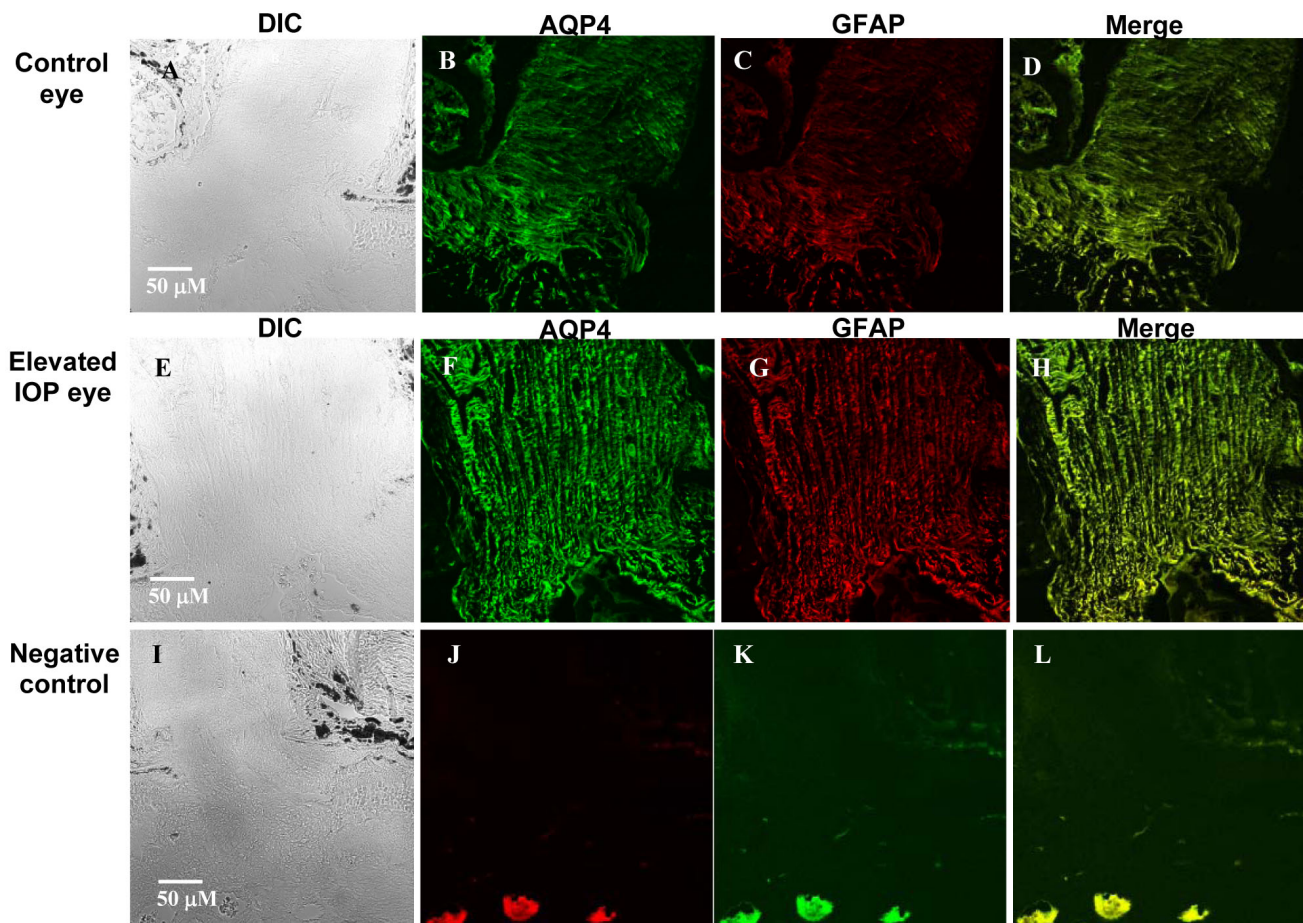
Expression of immunoreactive AQP4 (green) and glial fibrillary acidic protein (GFAP; red) in the optic nerve of rats exposed to experimentally elevated intraocular pressure. Elevation of IOP was performed as described by Morrison et al [22]. The contralateral (control) eyes showed modest AQP4 and GFAP expression (**B-D**). In contrast, AQP4 and GFAP labeling at the optic nerve was consistently more intense in eyes exposed to elevated IOP compared to control eyes (**F-H**). A representative figure of the optic nerve is shown for one rat exposed to >500 mmHg days of IOP elevation. **A** and **E** are DIC images, **B** and **F** represent AQP4 labeling, **C** and **G** represent GFAP labeling, and **D** and **H** represent merged images of AQP4 and GFAP labeling (yellow). Scale bars represents 50 μm. Staining was performed without primary antibodies (**I-L**) that shows no labeling and was used as a negative control.

Whereas AQP4 levels appeared to decrease in the retina, based on immunohistochemistry results, it was possible to detect qualitative changes in immunoreactive AQP4, and GFAP levels in the optic nerve of rats with elevated IOP. In the control group, immunostaining for AQP4 was visible in the three regions of the ONH (Figure 7). The pattern of distribution of AQP4 was similar to the pattern of GFAP-positive cells, suggesting that expression of AQP4 was located in astrocytes. In the group with chronic elevated IOP, AQP4 expression was also seen in the three regions of the ONH. A greater number of stained cells were visible as compared with those of the control group (Figure 7). Immunohistochemical studies on retinal sections of ET-1 intravitreally injected rats did not show significant changes in AQP4 compared to HEPES-injected eyes (data not shown).

**Figure 8:**
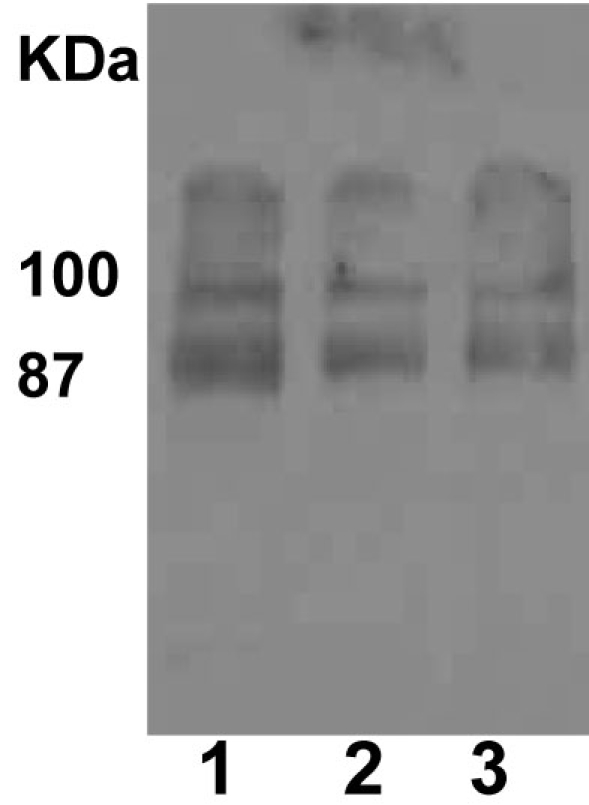
Detection of aquaporin-4 (AQP4) in isolated ubiquitinated protein fractions. S5a is a protein subunit of the proteasome that binds ubiquitinated proteins and can be used to purify ubiquitinated proteins. Retinal ubiquitinated proteins were purified on an S5a affinity column (Biomol Inc) before they were eluted with SDS-sample buffer. Next, they were separated on SDS-PAGE then subjected to western blotting using anti-AQP4 antibodies. As shown in this figure, higher molecular weight forms of AQP4 were detected (87 and 100 kDa proteins). Lanes 1-3 are three different retinal preparations from different rats.

**Figures 9:**
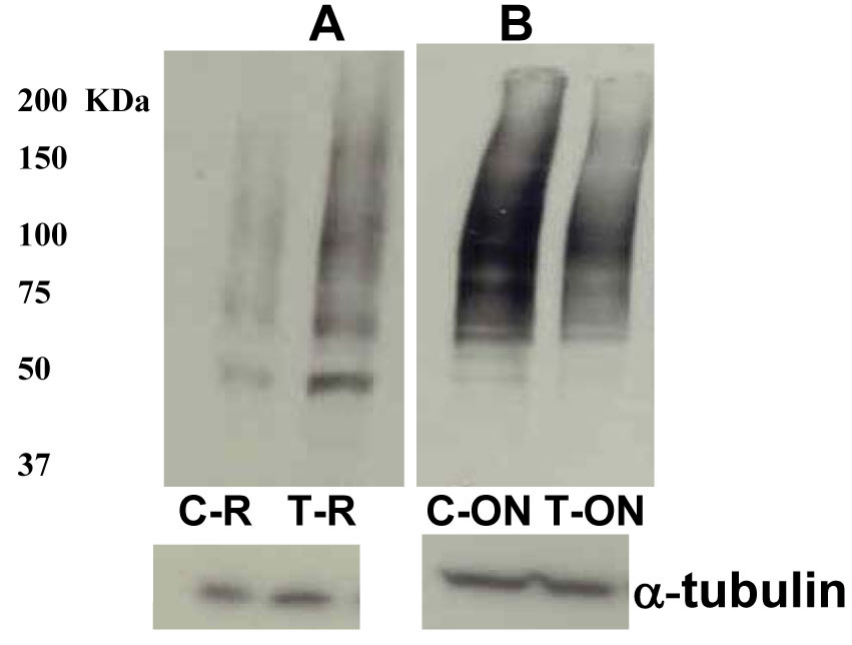
Elevation of IOP increased ubiquitination in retinal extracts and decreased ubiquitination in optic nerve extracts. Ubiquitination starts with monoubiquitination at multiple lysine residues then extending the ubiquitin chains by the sequential addition of multiple ubiquitin moieties and that is why when using the anti-ubiquitin antibodies, immunoreactive smear is detected that reflects the covalent attachment of multiple ubiquitin chains to individual protein molecule. Western blot with anti-ubiquitin antibodies revealed a characteristic ubiquitin-positive smear in each lane corresponding to the amount of ubiquitination on proteins, and elevation of IOP enhanced ubiquitination in retinal extracts (**A**) while decreased ubiquitination in optic nerve extracts (**B**). Abbreviations: C-R is control retina, and T-R is elevated IOP retina; C-ON is control optic nerve, and T-ON is elevated IOP optic nerve.

**Figure 10:**
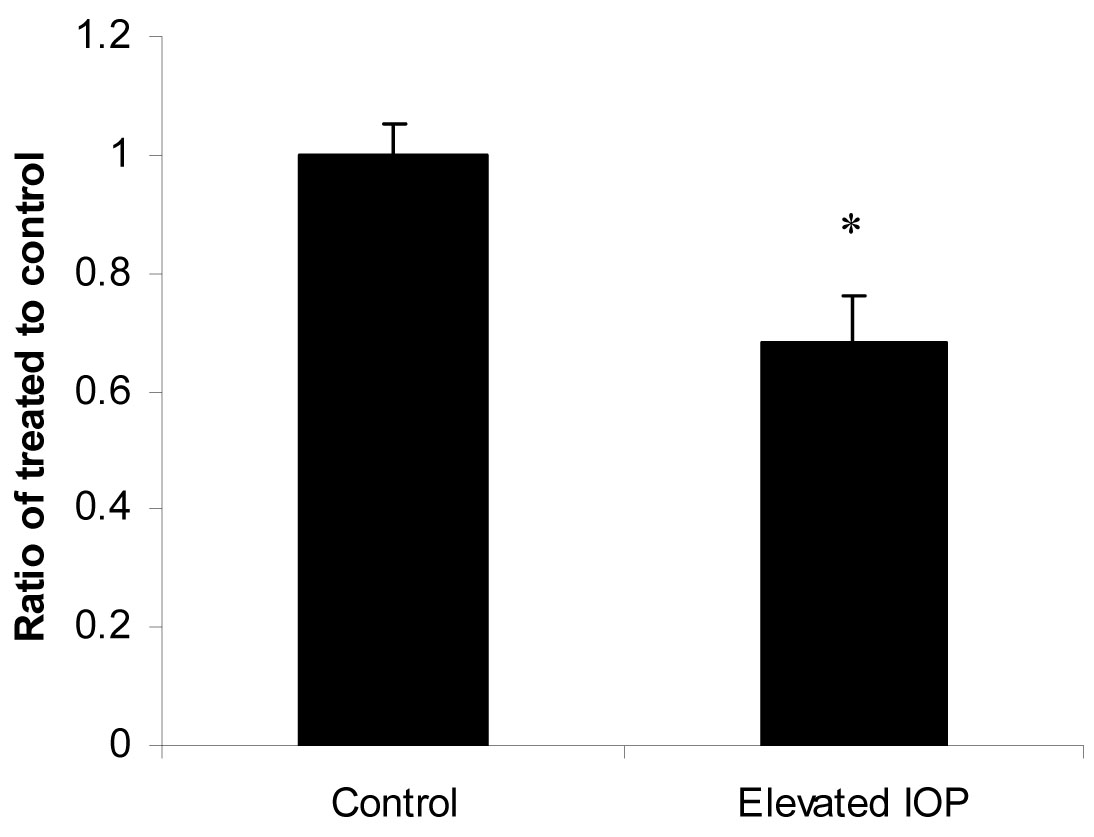
Elevation of intraocular pressure decreased UCH-L1 mRNA levels. Total RNA was isolated and transcribed into cDNA. Real-time PCR was performed using specific primers (see Methods). mRNA expression of UCH-L1 was adjusted to the mRNA copies of β-actin (reference gene). Results indicate that mRNA expression level of UCH-L1 was significantly lower in elevated intraocular pressure (IOP) retinas compared to control. UCH-L1 commonly linked to other neurodegenerative diseases, may play a role in optic nerve degeneration. Data are expressed as a ratio of the control value and each column represents mean±SEM. The asterisk denotes statistical significance of ratio from IOP treatment versus that of control as determined by One-way ANOVA and Tukey multiple comparison test at p<0.05.

The difference between retina and optic nerve AQP4 data could be explained due to differences in activities of the ubiquitin-dependent proteasome. Several channels have been shown to undergo ubiquitination and degradation. Affinity purified ubiquitinated proteins on an S5a column from rat retinas revealed the presence of 87 and 100 kDa AQP4 high-molecular weight isoforms, suggesting that AQP4 is a target for degradation by the ubiquitin-dependent proteasome (Figure 8). Levels of ubiquitination in both retinal and optic nerve protein extracts were analyzed by western blotting using monoclonal anti-ubiquitin antibodies. Although ubiquitination starts with monoubiquitination at multiple lysine residues, ubiquitination activity can extend the ubiquitin chains by the sequential addition of multiple ubiquitin moieties. This is why when the anti-ubiquitin antibodies are used, immunoreactive smear is detected that reflects the covalent attachment of multiple ubiquitin chains to individual protein molecule. Western blot with anti-ubiquitin antibodies revealed a characteristic ubiquitin-positive smear in each lane corresponding to the amount of ubiquitination on proteins, and elevation of IOP enhanced ubiquitination in retinal extracts while decreased ubiquitination in optic nerve extracts (Figures 9A,B, respectively, n=10). Enhanced ubiquitination may reflect accelerated degradation of proteins, including AQP4, which may explain the different effects of elevated IOP on AQP4 levels in retina versus optic nerve. Elevated IOP attenuated expression of UCH-L1 in retina as judged by Q-PCR (8 out 10 rats, 68±8%, p<0.005, Figure 10). Although the downregulation of ubiquitin hydrolase may explain the enhanced ubiquitination in retina, UCH-L1 protein levels did not change significantly in retinas with elevated IOP (data not shown). Overall, in both retinal injury models, both AQP4 and thy-1 levels were decreased in retinas. By contrast, AQP4 increased in ONH astrocytes, a mechanism that may involve alterations of the ubiquitin-dependent proteasomal activities. Also, both procaspase-3 and GFAP levels were increased following retinal injuries.

## Discussion

The role of AQP4 in glaucoma has not been fully understood. In the current study, elevation of IOP appears to affect AQP4 levels differently in the retina and ONH. While AQP4 decreased in retina, it increased in the ONH and colocalized with GFAP. These observations suggest that IOP elevation is an important factor for increasing the levels of AQP4 and GFAP, which could promote glial activation at the optic nerve. The upregulation of water channels in rat ONH with elevated IOP could lead to hypertrophy. Patients with normal tension glaucoma [34] as well as POAG [35] have a larger optic disc compared to normal patients. Whether the upregulation of AQP4 explains astrocyte hypertrophy in the ONH is not clear and may require a selective AQP4 knockout in ON astrocytes. The lack of selective AQP4 inhibitors also hampers a quicker evaluation of whether AQP4 inhibition will prevent hypertrophy upon elevation of IOP. More importantly, a new role for astrocytic AQP4 in the development of neuromyelitis optica (NMO) has been reported. NMO is an autoimmune disease characterized by severe optic neuritis and transverse myelitis where serum from patients is enriched with anti-AQP4 antibodies leading to loss of AQP4 in the active perivascular NMO lesions. GFAP was strongly stained at the reactive astrogliosis surrounding the lesions and therefore, it has been suggested that astrocytic impairment is associated with humoral immunity against AQP4 [36].

In addition, AQP4 deletion in mice is neuroprotective in a transient ischemia model of retinal injury [37] and hypoxia induced a marked decrease in AQP4 mRNA levels in astrocytes in vitro [38], suggesting that the reduction of AQP4 levels may be a beneficial mechanism. However, AQP4 also appears to play an important role in ion homeostasis and “K^+^ spatial buffering” [39–43]. Retinal water transport or changes in extracellular space volume appear to parallel transglial K^+^ currents, and mice lacking AQP4 have a significant delayed cellular reuptake of K^+^ from the extracellular space (ECS) [44–46]. K^+^ spatial buffering within the inner retina occurs via the redistribution (siphoning) of excess K^+^ from the extraneuronal space toward fluid reservoirs of low K^+^ (or sinks), such as vitreous body, subretinal space and blood vessels [47,48]. This is especially important since firing of neurons changes the extracellular concentration of K^+^ ions ([K^+^]_o_) due to excess K^+^ ions liberated from neurons into the intracellular space and causes a slow depolarization of glial cells which have the ability to maintain [K^+^]_o_ at a constant level. Spatial K^+^ buffering generates osmotic gradients leading to uptake of sodium and bicarbonate that causes the intracellular osmolarity to increase and drives water into the glial cells through AQP4. An imbalanced reduction of AQP4 levels may therefore impair retinal K^+^ buffering and uncontrolled increases in [K^+^]_o_ may induce uncontrolled hyperexcitability and abnormal synchronization of retinal neurons.

In addition, the expression of several aquaporins including AQP1, AQP2, AQP3, and AQP5 decrease with aging [49–51], and therefore AQP4 expression may also decrease with aging. Interestingly, human retinal glial cells display an age-dependent decline in their K^+^ conductance (an estimated 50% reduction), in cells from donors aged 60 years and more [52], and it remains to verify if such attenuation is linked to or parallels changes in AQP4 retinal levels.

The difference in affecting AQP4 protein levels may involve the ubiquitin-dependent proteasome. Western blot analysis detected high molecular weight isoforms of AQP4 and these appeared to be ubiquitinated isoforms. S5a-affinity purified ubiquitinated proteins were enriched in high molecular weight AQP4 isoforms. Increased IOP appeared to enhance ubiquitination in the retina but decreased it in the optic nerve. Elevated IOP decreased UCH-L1 mRNA levels in the retina, and this may also explain the increased ubiquitination in retina. Although such an observation may explain the different changes in AQP4 between the retina and optic nerve, other mechanisms cannot be ruled out. Bizzi et al. have shown that ubiquitin utilizes anterograde transport in the optic nerve axons exclusively with the slow component b (SCb), known to carry cytoskeletal and cytoplasmic proteins [21]. It is well documented that elevated IOP blocks both anterograde and retrograde axonal transport in the ONH in animals [2–7]. Therefore it is possible that the elevation of IOP would inhibit ubiquitin transport to axons and thus explain the attenuated ubiquitination in axons, whereas the accumulation of ubiquitin in retina could account for the enhanced ubiquitination and the downregulation of AQP4 protein. More important, the attenuation of ubiquitination in axons may result in accumulation of several proapoptotic proteins (e.g., caspases, Bax and Bad) known to be degraded by the proteasome [20] and thus contributes to axonal degeneration in glaucoma.

There are several studies that looked at similarities between glaucoma and other neurodegenerative diseases and our data found a common link. UCH-L1 has been implicated in many neurodegenerative diseases, such as gracile axonal dystrophy in mice, familial Parkinson disease [53–55], diabetic spiral ganglion cells in WBN/Kob rats [56], and lepromatous eyes [57]. The current finding that UCH-L1 mRNA levels are decreased with elevated IOP (Figure 10) is consistent with the findings in other neurodegenerative diseases. However, the involvement of other components of the proteasomal system may contribute as well but needs further investigation.

By western blot, we demonstrated an increase in GFAP protein levels using two different models of retina injury: intravitreal ET-1 injection and the Morrison model of glaucoma. Increased GFAP labeling was similarly observed at the rat ONH exposed to elevated IOP as described by Johnson et al. [58] and Prasanna et al. [59]. It is well known that a universal early cellular marker for retinal injury is the upregulation of the intermediate filament protein, GFAP. Although the exact function of GFAP is not known, its immunoreactivity is commonly used as an index of gliosis/hypertrophy [32,33]. In POAG patients, increased GFAP staining was observed in astrocytes at the ONH that correlated strongly with the severity of glaucoma [32,33]. Tanihara et al. [60] have shown that elevation of IOP in primates was accompanied by increased GFAP mRNA and Yu et al. [61] observed elevation of IOP increased GFAP protein levels without significant changes in GFAP mRNA levels. Ju et al. [62] demonstrated that chronic ocular hypertension was accompanied by increased GFAP expression in rats. Furthermore, Lau et al. have shown that intravitreal ET injection increased GFAP expression in rat retina [63]. Thus increased GFAP expression appears to accompany damage to the optic nerve and retina.

The present data are also in agreement with earlier studies that reported elevated IOP induced activation of caspase-3 [24,64–66]. Other groups have used axotomy and shown activation of caspases [67–69]. In the present study, western blot of caspase-3 detected the 31 kDa pro-caspase-3 form and not the active cleaved caspase-3 (17 kDa), although it has been suggested the antibody can recognize both species. The reason for that could be due to many factors. Johnson et al. [70] used the same antibody and detected only the procaspase-3 and not the cleaved fragment, and Garcia-Domingo et al. [71] used antibodies against caspase-3 and were able to detect procaspase-3 but not the cleaved fragment. However, McKinnon et al. [24] used antibodies from Idun Inc. and detected procaspase-3 and cleaved caspase-3. We tested the “marketed” anticleaved caspase-3 only from Cell Signaling, and we were surprised that it recognized the procaspase-3 and did not observe the 17 kDa fragment (data not shown). One explanation for the lack of detection of the cleaved caspase-3 fragment is its subsequent ubiquitination and degradation. Three studies have shown that the 17 KDa fragment is quickly ubiquitinated and degraded by the proteasome, which may explain its absence [70,72,73].

Also, similar to earlier reports [74–76] showing a reduction in thy-1 levels, elevation of IOP and intravitreal ET-1 injection resulted in decreased thy-1 levels (Figures 6A,B). Both Huang et al. and Schlamp et al. [74,75] reported a reduction in thy-1 levels very early following IOP that preceded the actual reduction of RGC number. Therefore, a reduction in thy-1 occurs in advance of detectable ganglion cell loss and both studies have suggested that thy-1 serves as an early marker of RGC stress but not a marker of RGC loss.

In summary, the current study reports a novel finding of increased expression of AQP4 in ON astrocytes that may be linked to cell hypertrophy. However, it remains to be verified that AQP4 inhibition or knockout may prevent or delay hypertrophy. This suggests that the levels of AQP4 in human glaucomatous eyes need further study. Increases in IOP appear also to affect AQP4 and ubiquitination levels distinctly in retina and optic nerve. The decreased ubiquitination in axons may increase the levels of proapoptotic proteins normally degraded by the proteasome and thus contributes to axonal degeneration following elevated IOP. Finally, similar to other neurodegenerative diseases, UCH-L1 may also be involved in IOP-induced retinal damage.
